# Stress memory and its regulation in plants experiencing recurrent drought conditions

**DOI:** 10.1007/s00122-023-04313-1

**Published:** 2023-02-15

**Authors:** Carolyn Mukiri Kambona, Patrice Ahossi Koua, Jens Léon, Agim Ballvora

**Affiliations:** 1 Department of Plant Breeding, Institut Für Nutzpflanzenwissenschaften Und Ressourcenschutz (INRES), RheinischeFriedrich-Wilhelms-University, Bonn, Germany; 2 Deutsche Saatveredelung AG, Thüler Str. 30, 33154 Salzkotten-Thüle, Germany; 3 Field Lab Campus Klein-Altendorf, Rheinische Friedrich-Wilhelms-University, Bonn, Germany

## Abstract

Developing stress-tolerant plants continues to be the goal of breeders due to their realized yields and stability. Plant responses to drought have been studied in many different plant species, but the occurrence of stress memory as well as the potential mechanisms for memory regulation is not yet well described. It has been observed that plants hold on to past events in a way that adjusts their response to new challenges without altering their genetic constitution. This ability could enable training of plants to face future challenges that increase in frequency and intensity. A better understanding of stress memory-associated mechanisms leading to alteration in gene expression and how they link to physiological, biochemical, metabolomic and morphological changes would initiate diverse opportunities to breed stress-tolerant genotypes through molecular breeding or biotechnological approaches. In this perspective, this review discusses different stress memory types and gives an overall view using general examples. Further, focusing on drought stress, we demonstrate coordinated changes in epigenetic and molecular gene expression control mechanisms, the associated transcription memory responses at the genome level and integrated biochemical and physiological responses at cellular level following recurrent drought stress exposures. Indeed, coordinated epigenetic and molecular alterations of expression of specific gene networks link to biochemical and physiological responses that facilitate acclimation and survival of an individual plant during repeated stress.

## Introduction

Global warming is one of the most important effects of climate change because it poses the heaviest environmental challenge confronted by mankind at the moment (Rajak 2021). It is not only influencing the air temperature but is also affecting the amount and distribution of precipitation, thereby resulting to future more frequent drought spells (Wang et al. 2014). Drought stress has been reported as one of the most destructive abiotic stress factors globally and generates a huge negative impact on crop production (Vurukonda et al. 2016; Koua et al. 2021). In describing agricultural drought, Trenberth et al. (2014) relate it to deficit in moisture in the topmost of about one meter of soil usually the root zone, thereby impacting crops. A meta-analysis of data collected between 1980 and 2015 showed that drought stress led to 40% yield reductions in maize and 21% yield reductions in wheat (Daryanto et al. 2016). Between the years 2005 and 2015, economic loses induced by drought were estimated to be around 29 billion USD (Trenberth et al. 2014; F.A.O. 2021). Recent droughts have had strong impact on world cereal production and will continue to cause year to year yield fluctuations (F.A.O. 2021), with predictions of having 50% of arable land under drought stress by the year 2050 (Kasim et al. 2013).

Drought stress can occur in every growth stage of a plant and influence the water relations of the plant at all levels including whole plant, organs, cellular and molecular levels (Li et al. 2014; Muscolo et al. 2015). In general, the growth and development of a plant are affected, thereby resulting to production of smaller organs as well as altered production of flowers and grain filling (Farooq, et al. 2009). In addition, stomatal closure is followed by a progressive decline in net photosynthetic activity and water-use efficiency, which greatly impair the productivity of plants (Wu et al. 2022).

Different from other organisms, plants are rooted permanently to one location and only respond to environmental cues through adjustment of growth and development patterns. Thus, flexibility is an essential requirement for plants to survive stress, which they maintain through operation of a signal response network (Amtmann & Armengaud 2009; Cutler et al. 2010) that enables them to reprogram their molecular machinery including transcription factors, stress-responsive proteins and secondary messengers (Tani et al. 2019). Plants also respond to drought by adjusting their metabolism/biochemical machinery like ethylene, proline and auxins alterations (Nair et al. 2008; Sharma et al. 2012). In addition, physiological changes involving cell membrane stability and osmotic adjustment (Abid et al. 2018), and morphological changes (phenotypic plasticity) (Basu et al. 2016) occur in plant during exposure to drought.

Recently, researchers have discovered that the ability of plants to adjust response mechanisms in a continuously changing environment shapes their fitness in future and eventually enables them to live in highly diversified habitats (Fleta-Soriano and Munné-Bosch 2016). Upon exposure to stress, plants alter their epigenetic, physiological and metabolomics machineries that modify responses to future similar stress in the same generation (somatic) and/or in the next generation(s) (intergenerational or transgenerational) to adapt and survive in many ways. This popular phenomenon in which an environmental signal prepares a plant for possible future stress exposure is referred to as priming. Xin and Browse (2000) described it as a resource saving strategy of improving plant tolerance to stress. The preservation of a primed state over time forms the basis of stress memory (Haider et al. 2021). Regardless of what plant’s future holds, the first stress exposure will leave an imprint in the plant that affects how it responds to later stresses (Liu et al. 2021a). Therefore, stress memory in plants is the capability of a plant upon exposure to stressors to store stress information so that it can respond in a different fashion when challenged by the same stress later (Bruce et al. 2007; Avramova 2015; Bilichak et al. 2015; Crisp et al. 2016; Fleta-Soriano and Munné-Bosch 2016). This capability is an integral part of plant resilience under changing climate.

Available studies exploring the topic of stress memory in plants have so far advanced the understanding of priming by detailing epigenetic, transcriptional, proteomic and physiological alterations resulting to imprints that establish stress memory in plants (Liu et al. 2022a; Sharma et al. 2022; Singh & Prasad 2022). While these studies have described variation between epigenetic marks and their effect on stress response, the integration of altered gene expression due to these modifications with physiological, biochemical and morphological responses of plants during recurrent stress is not well explored. We elucidate the interconnection of these mechanisms during recurrent drought episodes by describing the coordinated stress memory changes (imprints) at different OMICS, cellular and organismal levels that prepare plants to be more responsive to future stress within or across generation(s), which could provide new opportunities for crop improvement to ensure food security (Fleta-Soriano and Munné-Bosch 2016; Godwin & Farrona 2020).

In this review, we (1) classify stress memory in plants and give an overall view using general examples; (2) focus on drought stress and summarize the epigenetic modifications associated with gene expression control during recurrent drought episodes; and (3) correlate transcriptional and posttranscriptional memory with various drought memory imprints.

## Classifications of stress memory based on time point of stress and mode of inheritance

**Fig. 1:**
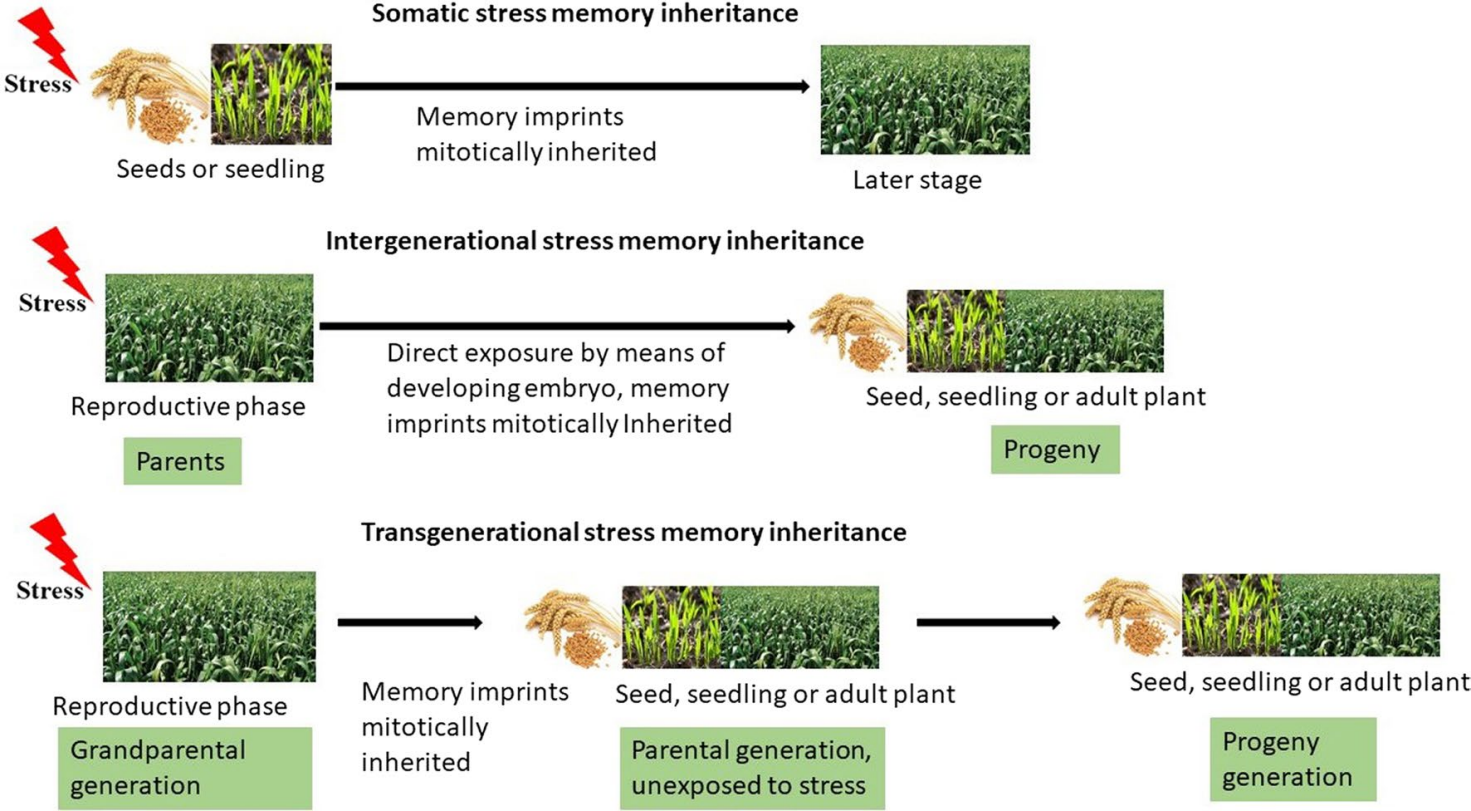
Somatic, intergenerational and transgenerational stress memory. Memory is dependent on stage of the plant at which priming is done

Various terms have been devised to describe the different stress memory types, usually based on the stage of the plant when priming is done and the mode of inheritance (Fig. 1).

### Somatic stress memory

Stress memory that is limited to one generation in duration is referred to as somatic stress memory (Lämke and Bäurle 2017). While the abiotic stresses occurring at different stages result in a higher risk of injury, plants can experience stress at an early stage during their growth and development, which can induce short-term stress memory to allow the plants to be tolerant if a similar stress strikes in later developmental stages (Li and Liu 2016). Therefore, somatic stress memory lasts for a short period of time, and its memory imprints are inherited mitotically.

### Intergenerational versus transgenerational stress memory

Exposing a plant (parental generation) to drought stress during the reproductive phase also exposes its reproductive cells and the resulting seeds to the same drought stress. Therefore, stress memory in the progeny generation could be mediated by cues introduced into the seed or embryo by the parental plant. This type of stress memory is referred to as intergenerational and implies the direct exposure to the stressor of the parental generation and the following generation (progeny) by means of the developing germ cells (Heard & Martienssen 2014; Lämke & Bäurle 2017).

On the other hand, transgenerational transmission is present when effects of the ancestral exposure to an environment during reproductive stage are present in the generation that is not directly exposed (Klengel et al. 2016). Hence, if grandparental generation was exposed to stress at reproductive stage, true transgenerational inheritance can only be observed in the progeny generation, when the parental generation had been unexposed (recovery period) (Fig. 1).

## Stress memory to various stressors in plants

Whether plants can remember is a provocative question that has lately preoccupied scientists. Recent studies addressing priming and stress memory have provided new valuable evidence on responses that are key factors of priming induced stress tolerance (Table 1).

**Table 1:** Stress memory development in different crop plants

Stressor	First encounter—when?	Recurrent encounter(s)—when?	Plant species	Memory imprints	Reference(s)
*Pseudomonas syringae* pv *tomato* DC3000 (*Pst*DC3000)	Seedling stage	Reproductive stage	Arabidopsis (*Arabidopsis thaliana* L.)	Activation of salicylic acid (SA)-inducible defense gene	(Luna et al. 2012)
Herbivores—*Pieris rapae*	Seedling to harvest	Seedling stage	Radish plants (*Raphanus raphanistrum* L.)	Increased seed mass, early plant growth	(Agrawal 2002)
*Tobacco mosaic virus* (TMV)	Seedling to harvest	Seedling stage	Cultivated tobacco (*Nicotiana tabacum* L.)	Smaller and few lessions	(Roberts 1983)
*Brevicoryne brassicae*	Pre- to early flowering	Post-flowering and late flowering	Rapeseed (*Brassica napus* L.)	Increased levels of glucosinolate	(Lammerink et al. 1984)
*Turnip mosaic virus *(TuMV)	8th day of growth	14th day of growth	Mustard (*Brassica campestris* L.)	Increased glucosinolate concentration	(Shattuck 1993)
Carbon dioxide (CO_2_) and soil nitrogen (N)	Reproductive stage for 5 seasons	Seedling stage	Sundial lupine, meadow grass and little bluestem (*Lupinus perennis L., Poa pratensis* L. and *Schizachyrium scoparium* L.)	Increased biomass and growth	(Lau et al. 2008)
Carbon dioxide (CO_2_)	Reproductive stage	Seedling stage	*Red brome * (*Bromus rubens* L.)	Altered nitrogen dynamics, Reductions in photosynthesis and growth rates	(Huxman et al. 2001)
Heat/ high temperature	Bolting stage till harvest	Bolting stage	*Arabidopsis thaliana* L	Improvement in fitness (increased seed production per individual)	(Whittle et al. 2009)
Salt stress	Reproductive stage	Reproductive stage	*Arabidopsis thaliana* L	Higher expression of AtRad51, higher tolerance to salt, bigger rosette and early flowering plants	(Boyko et al. 2010; Groot et al. 2016)
Physical disturbance			Sensitive plant (*Mimosa pudica L.*)	Leaf-folding habituation, memory of inhibitory modifications and recall	(Gagliano et al. 2014)
Salt (NaCl)	Seeds	seedling	*Rapeseed* (*Brassica napus L*.)	Higher total emergence and dry weight, enhanced proline accumulation	(Farhoudi et al. 2007)
Salt (NaCl)	Seeds	Seedling	Rapeseed(*Brassica napus* L.)	Higher total emergence and dry weight, enhanced proline accumulation	(Farhoudi et al. 2007)
Low Temperature	Seeds	Seedling stage	Okra (*Abelmoschus esculentus* L.)	Increased membrane integrity	(Dkhil et al. 2014)
Heat	Before anthesis	After anthesis	Common wheat (*Triticum aestivum* L.)	Up-regulated the Rubisco activase B encoding gene *RcaB,* higher photosynthesis rate	(Wang et al. 2014)
Salt	Seedling stage	Seedling stage	Maize (*Zea mays* L.)	Increased proline levels	(Tajdoost et al. 2007)
Seed priming with water, CaCl_2_, moringa leaf extracts and salicylic acid	Seeds	Seedling stage	Maize (*Zea mays* L.)	Reduced the electrical conductivity, increased the leaf relative and chlorophyll contents, increased plant height and yields	(Rehman et al. 2015)
Salt	Seedling stage	Seedling stage	Sorghum (*Sorghum bicolor* L.)	Higher photosynthetic rate, enhanced osmotic resistance and reduction in root Na + uptake	(Yan et al. 2015)

Intensive research has been conducted to study pre-exposure of plants to biotic and abiotic stressors, which trigger stress memory response. These memory imprints enable the plants to be ready to respond to subsequent stressful events (Xin & Browse 2000; Luna et al. 2012; Balmer et al. 2015; Hossain et al. 2015; Wang et al. 2017, 2018; Fan et al. 2018; Leuendorf et al. 2020). For instance, Agrawal (2002) found out that destruction of *Raphanus raphanistrum* L. following attacks from *Pieris rapae* L. during the vegetative phase of growth had influenced the induction of resistance on progeny in a later attack when compared to the controls and additionally reported that herbivory in the maternal generation influenced the growth of the progeny especially on seed mass. Furthermore, other studies in the past had indicated the possibility of memory from attacks by aphids, pathogens and other predators, thereby portraying induced resistance on later attacks (Rogers 1966; Roberts 1983; Lammerink et al. 1984; Shattuck 1993).

In a study on three different plant species that had been grown under two CO_2_ concentrations, Lau et al. (2008) discovered that the maternal CO_2_ environment during grain filling stage influenced biomass of progeny of all the species. The elevated carbon dioxide (eCO_2_) memory increased growth response to a future eCO_2_, a finding that had been contradicted by Huxman et al. (2001), who by using *Bromus Rubens* L. found out that the effects of maternal exposure to eCO_2_ reduced the performance of the progeny grown under eCO_2_ treatment especially by reducing photosynthesis and growth rates.

Whittle et al. (2009) assessed stress memory to find out if *Arabidopsis thaliana* L. plants adaptively responded to environmental conditions experienced by their ancestors. They examined plants that were exposed to mild heat or cold environments in parental and F_1_ generation and discovered that previous elevated temperature treatment led to a more than fivefold improvement in fitness in F_3_ generation. After checking the persistence of previous stress memory, they reported that improvement due to heat memory in F_3_ generation plants remained even when the heat-exposed parental and F_1_ plants were grown in a normal temperature regime in F_2_ generation. Using *Arabidopsis thaliana* L., the ability of plants to remember salt stress exposure as far as four generations ago was found, and transgenerational as well as somatic effects in almost all analyzed traits were observed (Groot et al. 2016). Similarly, Boyko et al. (2010) had reported that transgenic *Arabidopsis thaliana* L. offspring from salt stress-exposed parents showed increased tolerance to salt and had higher rates of recombination.

In an experiment conducted by Gagliano et al. (2014) using a sensitive *Mimosa pudica* L. plant*,* whose leaves close rapidly by folding to respond to mechanical disturbance, it was interesting to realize that when the plant was initially dropped to experience mechanical stress, the leaves reacted by closing tight. However, when they dropped the plant repeatedly, its response changed and did not react as expected but the leaves stayed open. This was a clear indication of training and adaptation that suggested learning and memory mechanisms. The authors also noted that the sensitive plants displayed the learned response also when they were placed for a month in a favorable environment without disturbance. In animal studies, memory is considered as long term if one can store information for 24 h and remember (Sánchez-Andrade and Kendrick 2011). Therefore, based on rules routinely used, the mimosa plants had shown that they were capable of learning and remembering what they had learnt.

## Drought stress memory as a mechanism of plant adaptation

Plants’ responses to drought stress have been widely investigated because drought can occur at any stage of growth, from vegetative to grain filling, thereby negatively influencing yield production. Among other mechanisms of adaptation and tolerance to water scarcity, various studies have demonstrated drought stress memory in several species (Table 2) like in *Brassica napus* L. (Hatzig et al. 2018), *Trifolium repens* L. (Rendina González et al. 2018), wheat (Liu et al. 2020), rice (Zheng et al. 2013), *Polygonum persicaria* L. (Herman et al. 2012), *Arabidopsis thaliana* L. (van Dooren et al. 2020), *Leontodon hispidus* L.*, Plantago lanceolata* L. and *Trifolium pratense* L. (Cerda 2020), suggesting that previous drought stress exposure left some stress imprints that were stored to induce improvement in a subsequent stress encounter.

**Table 2:** Summary of studies tackling repeated drought stress in various crop species. DAS, days after sowing; FC, field capacity

Plant species	Initial stress	Repeated stress	Memory type	Memory imprints	Reference(s)
Sugarcane (*Saccharum officinarum* L.)	Withdrawal of water	Two more cycles of water withdrawal and recovery, propagules were subjected to water-deficit	Somatic	Faster recovery of CO_2_ assimilation and higher instantaneous carboxylation efficiency	(Marcos, Silveira, Marchiori, et al., 2018a)
Oat (*Arrhenatherum elatius* L.)	Early drought stress followed by rewatering	Later drought stress	Somatic	High living biomass, improved photoprotection	(Walter et al. 2011)
Sugarcane (*Saccharum officinarum* L.)	First water-deficit cycle	Second and third water-deficit cycles	Somatic	Increases in intrinsic water-use efficiency, higher root water concentrations	(Marcos et al. 2018b)
Potato (*Solanum tuberosum* L.)	50% FC at tuber initiation stage	Second stress like the first	Somatic	Higher tuber yields, increased antioxidant activity	(Ramírez et al. 2015)
Barley (*Hordeum vulgare* L.)	Withholding of water at full flag leaf stage (BCH 45–47) followed by rewatering	Withholding of water at seedling stage	Transgenerational	Increased thin roots and seed-derived nutrients	(Nosalewicz et al. 2016)
Durum wheat (*Triticum durum* L.)	withholding water and rewatering to field capacity	Withholding of water following the rewatering phase	Somatic	Activated oxygen production and detoxification	(Menconi et al. 1995)
*Arabidopsis* thaliana L	amino acid b aminobutyric acid (BABA) treatment by soil drench to 5-week-old plants	Drought applied one day after	Somatic	Earlier and higher expression of the salicylic acid-dependent PR-1 and PR-5 and the abscisic acid (ABA)-dependent RAB-18 and RD-29A genes	(Jakab et al. 2005)
*Arabidopsis thaliana* L	Removing the plants from soil and air-drying for 2 h	Recovery—plants placed 22 h in humid chambers for followed by a similar drought treatment	Somatic	An increase in the rate of transcription and elevated transcript levels of a subset of the stress–response genes	(Ding et al. 2012)
Common wheat (*Triticum aestivum* L.)	21 days after sowing, mild drought then rewatering for 48 h	Severe drought immediately after rewatering	Somatic	Induction of coordinated antioxidant defense, reduced H2O2 accumulation and membrane damage, higher relative water content	(Selote & Khanna-Chopra 2006, 2010)
Common wheat (*Triticum aestivum* L.)	Withdrawal of water 5–7 days at vegetative stage	Water withdrawal at grain filling stage	Somatic	reduced photoinhibition in flag leaves, higher concentration of abscisic acid	(Wang et al. 2015)
Ice plant (*Aptenia cordifolia* L.)	Withholding water for 10 days and rewatering for 4 days	Withholding water for 9 days,	Somatic	Increased abscisic acid	(Fleta-Soriano et al. 2015)
Common wheat (*Triticum aestivum* L.)	Soil relative water content around 35–40% before anthesis	Soil relative water content around 20–25%) 15 d after anthesis	Somatic	Higher photosynthesis rate and ascorbate peroxidase activity, altered protein expression	(Wang et al. 2014)
Rice (*Oryza sativa* L.)	Water withdrawal for 6 days followed by 3 days of rewatering	3 days Water withdrawal immediately after rewatering	Somatic	Low MDA, increased peroxidase (POD) and superoxide dismutase (SOD) activities, lncRNA, DNA methylation and endogenous phytohormones	Li et al. 2019; Li et al. 2011)
Orange (*Citrus sinensis L*.)	Withdrawal of water after two years of growth followed by rewatering	Two more subsequent cycles of drought stress	Somatic	epigenetic and hormonal (abscisic acid, auxins and salicylic acid) changes	(Neves et al. 2017)
*Arabidopsis thaliana* L	Removal of plants from soil and air-drying for 2 h, then rehydration by dripping water to the root for 24 h	Dehydration was repeated	Somatic	Changes in the distribution level of AhATL1expression and AhATL1	(Qin et al. 2021)
Beet (*Beta vulgaris* L.)	Water withdrawn 35–54 DAS, followed by rewatering	86–102 DAS, rewatering, 135–151 DAS, rewatering	Somatic	Alterations in osmotic potential, proline and chlorophyll content	(Leufen et al. 2016)
Soybean (*Glycine max* L.)	Water-deficit at 4-day-old seedlings, recovery	Two more drought phases with recovery in between	Somatic	changing of biochemical parameters (soluble sugar and proline)	(Nguyen et al. 2020)
Common nettle (*Urtica dioica* L.)	Water withdrawal at 49 DAS for 14 days, rewatering	Water withdrawal after flowering	Somatic	Increases in lipid peroxidation	(Oñate et al. 2011)
Rapeseed (*Brassica napus* L.)	Water withdrawal at reproductive stage till harvest	Water withdrawal at seedling stage	Intergenerational	Increased seedling fresh weight and concentrations of several amino acids and nitrogen compound	(Hatzig et al. 2018)
White Clover (*Trifolium repens* L.)	Water withdrawal with rewatering	Water withdrawal with rewatering, 7 more cycles	Intergenerational	Epigenetic change–DNA methylation alterations	(Rendina González et al. 2018)
Durum Wheat (*Triticum durum* L.)	Water-deficit stress was applied from the booting stage		Intergenerational	Differences in microRNA (miRNA) expression	(Liu et al. 2020, 2021b)
Rice (*Oryza sativa* L.)	Water withdrawal from tilling stage to seed filling stage	Water withdrawal from tilling stage to seed filling stage for another five generations and 10 generations	Intergenerational	Changes in DNA methylation patterns, transgenerational epimutations	(Zheng et al. 2013, 2017)
Redshank*—Polygonum persicaria*	Dry soil at seedling for 71 days	Achenes collected and allowed to grow to maturity under dry soil, offspring grown in dry soil	Somatic, intergenerational, transgenerational	longer root systems, increased biomass, greatest provisioning	(Herman et al. 2012)
*Arabidopsis thaliana*	30% soil moisture content at vegetative stage	30% soil moisture content at vegetative stage	Somatic and intergenerational	Changes in phenotypic, gene expression and DNA methylation	(van Dooren et al. 2020)
Soybeans—*Glycine max* L	80, 60, 40 and 20% replacement of evapotranspiration in reproductive stage		Somatic	Reduced germination, seedling vigor and seed quality	(Wijewardana, Raja Reddy, et al., 2019)
Common wheat (*Triticum aestivum* L.)	Tillering or jointing	Post-anthesis	Somatic	Improved leaf water potential, more chlorophyll and ribulose-1, 5-bisphosphate carboxylase/oxygenase contents, enhanced photosynthesis, better photoprotection and efficient enzymatic antioxidant system	(Abid et al. 2016)
Common wheat (*Triticum aestivum* L.)	Polyethylene Glycol (PEG) stress induction at seedling stage	Water withdrawal at jointing stage	Somatic	Physiological and biochemical changes	(Abid et al. 2018)
Rice (*Oriza sativa* L.)	Vegetative stage	Reproductive stage	Somatic	Proteome changes	(Auler et al., 2021b)
Potato (*Solanum tuberosum* L.)	One-month-old plant	One day after priming	Somatic	Photosynthesis, signal transduction, lipid metabolism, sugar metabolism, wax synthesis, cell wall regulation, osmotic adjustment	(Chen et al. 2020)
Soybean (*Glycine max* L.)	Withholding of water 7-day-old plants, rewatering	One day after priming	Somatic	Induction of drought stress memory genes	(Kim et al. 2020)
Rice (*Oriza sativa* L.)	Air-drying of 4-week-old plant for 80 min at 28 ^◦^C	22 h after priming Air-drying: 80 min at 28 ◦C and a similar repeat	Somatic	Regulation of alternative splicing events	(Yang et al. 2020)
Common Grapevine (*Vitis vinifera* L.)	Water reduced to 40 field capacity	Water reduced to 40% field capacity for 3 more seasons and then, withdrawn until complete leaf abscission in the 5^th^ season	Somatic	Reduction of xylem hydraulic safety margin	(Tombesi et al. 2018)

While on the one hand drought stress memory is viewed from an evolutionary perspective as an effective strategy that could prepare a plant for later stress by improving the plant’s potential for local acclimation to changing environments, some studies have nevertheless associated it with negative effects like delayed growth and development and reduced yield (Skirycz & Inzé, 2010; Crisp et al. 2016; Wijewardana et al. 2019a, b). Therefore, although mechanisms of drought stress memory could have evolved as adaptive approaches to enhance resistance against drought, the overall performance may be compromised, thereby leading to tradeoffs between yield and stress survival (Godwin & Farrona 2020).

## Molecular mechanisms controlling stress memory in plants

**Fig. 2:**
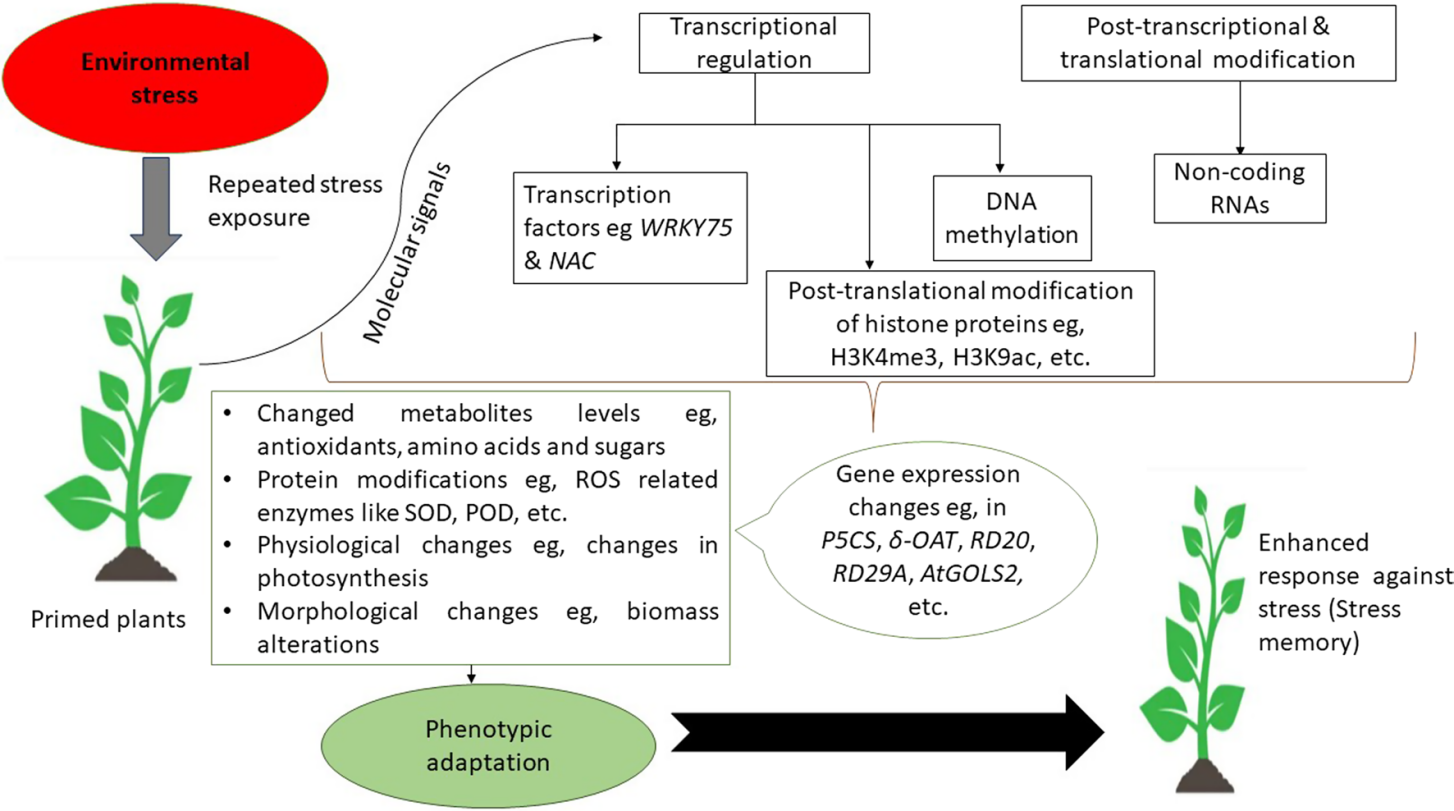
A graphic presentation of interactions between gene expression control during repeated exposure and stress responses. Inheritance of epigenetic regulators like histone modifications and DNA methylation, and the alteration of regulatory RNAs and transcription factors affect the expression of genes, thereby causing changes in phenotypes of the plant

Efforts are made to understand the mechanistic basis of stress memory. Liu et al. (2022a) emphasize that investigations on drought stress memory suggests that regulatory mechanisms on the transcriptional level vary in response to a single stress stimulus and repetitive stress stimulations. Several exposures to drought stress enable plants to respond to a new stress by more rapid adaptive changes to gene expression patterns compared with plants not previously exposed to a drought stress (Li and Liu 2016). Growing evidence points to a stress memory that might involve the maintenance of the response to stress by transcriptional, translational or epigenetic (DNA methylation and Histone modifications) means as summarized in Fig. 2 (Sousa et al. 2022). Epigenetic modifications are either mitotically or/ and meiotically heritable alterations in gene expression, which are independent of primary DNA sequence changes and potentially affect the outcome of a chromosome or locus without changing the underlying DNA (Bird 2007). According to Godwin and Farrona (2020), DNA methylation and histone modification constitute epigenetic marks within chromosomes that stably change gene expression and other chromosomal properties. Over recent years, it has become increasingly evident that transcriptional regulation cannot be fully understood unless the structural context in which it occurs is considered. Moreover, by frequently influencing the distribution of epigenetic marks, noncoding RNAs can act in a sequence-specific manner to regulate gene expression both at transcriptional and posttranscriptional levels, therefore playing an important role in epigenetic control (Thiebaut et al. 2019). On the other hand, regulation of transcription is a result of the combined effects of chromatin structural properties and the interaction of transcription factors. The transcriptional regulation by transcription factors (TFs) is the major step for the establishment of the gene expression network and has been implicated in the control of stress memory (Crisp et al. 2016). Therefore, we summarize the current findings on gene expression regulation mechanisms associated with drought stress memory by showing their integration with drought memory-responsive genes.

### Epigenetic regulation of transcription

#### Histone modifications and drought memory

DNA is in eukaryotes complexed with eight positively charged histone proteins, consisting of two molecules of each histone (H2A, H2B, H3 and H4), wrapped by 147 negatively charged DNA base pairs to make a nucleosome (Cutter & Hayes 2015). Generally, H2A, H2B, H3 and H4 can undergo covalent modification mostly at lysine and arginine residues by methylation, acetylation, ubiquitination, phosphorylation, biotinylation and ADP-ribosylation (Feng and Jacobsen 2011). Histone marks are a type of chromatin modifications that have been associated with drought-responsive memory genes and the subsequent enhancement of transcriptional response to recurrent drought stress. Kim et al. (2012) found a clear enrichment of H3K4me3 in the coding regions of drought-responsive genes *RD20*, *RD29A* and *AtGOLS2* that increased in response to drought stress and was maintained after gene deactivation by rehydration. In contrast, although H3K9ac increased initially during drought stress, it quickly responded to gene deactivation by rehydration and was drastically reduced on drought-inducible genes. This suggests the possibility of H3K4me3 to function as a stress memory epigenetic mark.

During repeated drought exposures on *Arabidopsis thaliana* L., even though the *RD29B* and *RAB18* genes returned to their initial non-stressed transcript levels when the plants were rewatered, they remained associated with uncommonly high levels of H3K4me3 and Ser5P polymerase II, demonstrating that RNA polymerase II is delayed or hindered in its activity (Ding et al. 2012). This observation supports the findings by Kim et al. (2012) regarding H3K4me3 as a drought stress memory epigenetic marker. The concept of transcriptional memory was clearly illustrated by the observed return of transcript levels to baseline during recovery and a higher induction of transcript levels on a subsequent stress exposure. In *Gossypium hirsutum* L.*,* Tian et al. (2022) revealed that H3K4me3 is necessary for the upregulation of memory genes *GhNCED9*, *GhPYL9-11A*, *GhP5CS1* and *GhSnRK2* during repeated drought, and its level on these genes decreased considerably on the 5th day following recovery. Memory genes with enriched H3K4me3 have also been documented, especially in *P5CS1* in salt stress and *HSP22.0* in heat stress (Feng et al. 2016; Lämke et al. 2016).

#### DNA methylation and drought memory

DNA methylation is an epigenetic modification where unlike in histone methylation, methylation unvaryingly takes place at the carbon-5 position of cytosine residues (Feng and Jacobsen 2011). Under the action of methylase, the DNA sequence of genes is not altered, but gene function is changed in response to external environmental stimuli. Generally, demethylation events are accompanied by the activation of genes, while methylation in the regulatory or coding regions hampers the expression of target genes (Sousa et al. 2022). This alteration is usually inherited by future generations to form epigenetic memory, which offers the possibility of breeding new crop varieties that are stress-tolerant.

Selfed progenies of drought-stressed plants showed increased DNA demethylation levels in *P5CS* and *δ-OAT* genes under subsequent drought than under control treatments. This clearly indicated that proline accumulation during repeated drought is facilitated by DNA demethylation, thereby upregulating the expression of these genes. The stability of DNA demethylation of these genes was observed through the increased proline accumulation in both onetime and two-time stressed plants growing under control environment, and subsequent higher levels of gene expression (Zhang et al. 2013).

Examination of the role of DNA methylation variations on rice adaptation to successive drought stress revealed non-random appearances of drought induced epimutations (Zheng et al. 2017), which was consistent to earlier findings that showed the induction of site-specific DNA methylation (Zheng et al. 2013). The authors noted that drought induced DNA methylation alterations were inherited in advanced generations and the genes associated with the discovered transgenerational DNA methylation changes were directly involved in drought-responsive pathways. Based on the Gene Ontology analysis of the non-TE genes related to both transgenerational and recurring DNA methylation alterations, their products are involved in signal transduction, development of flowers and pollination among others. For example, *LOC_Os08g33720* gene encoding a putative lactate/malate dehydrogenase and responding to abiotic stimuli, was found to have 12 hypo-methylated CG-DMPs with recurrence frequencies (Zheng et al. 2017).

The relationship of the expression of memory genes with differentially DNA methylated regions exposed that 5373 drought memory transcripts might be regulated by DNA methylation (Li et al. 2019). Kou et al. (2021) went ahead to examine how DNA methylation is involved in drought stress memory in rice cultivars under recurrent drought stresses and recovery treatments. The study confirmed that the identified differentially methylated regions (DMRs) mediate tolerance by gene expression and transposable elements regulation. Memory (DMRs) were found in promoter region of *LOC_Os05g38150* and in gene body of *LOC_Os01g62900* to directly regulate rice drought memory genes (Kou et al. 2021). Drought in the vegetative stage altered global DNA methylation levels in rice guard cells, and these modifications remained when drought was recurrent in the reproductive stage due to greater genomic stability at this stage (Auler et al. 2021b). Gene expression analysis in this study revealed that protein abundance had a positive correlation with the expression of their coding genes. Neves et al. (2017) revealed alterations in the global DNA methylation patterns that corresponded to an increase in ABA levels in citrus plants that were subjected to three cycles of drought when compared to plants that had experienced drought stress for the first time. However, a different study that investigated DNA methylome changes in *Arabidopsis thaliana* L. plants and five successive generations subjected to drought stress failed to link the transgenerational memory to epigenetic methylation (Ganguly et al. 2017). Taken together, much evidence indicates a prominent function of chromatin-based mechanisms in transcriptional memory responses linked to drought stress (Godwin & Farrona 2020).

### Regulatory RNA and drought memory

Small RNA molecules or microRNAs (miRNAs) are created from intergenic regions, repetitive sequences, transposable elements (TEs) and pseudogenes, accounting for more than 90% of all RNA transcripts (Nguyen et al. 2022). They regulate gene expression in signaling and other developmental pathways. According to Melnyk et al. (2011), systemic movement of drought triggered small RNAs through the symplast and vascular tissues to the meristem leads to DNA methylation by the RNA-directed DNA methylation (RdDM) pathway. Drought stress has been reported to induce expression of miRNAs to suggest their potential use in improving tolerance of plants. Guedes et al. (2018) performed miRNAs expression during the different cycles of drought stress on *Coffea canephora* L. and identified 198 miRNAs (21-nt sequences), from which most targets transcription factors (TFs). Based on differential expression analysis, *miRNA miR408* and *miR398* were highly up-regulated in the different drought stress cycles. Liu et al. (2020) uncovered differences in microRNA (miRNA) expression following repeated drought episodes, whose targets have critical molecular roles in stress adaptations. Liu et al. (2021b) have also reported the association of small RNA and their targets with transgenerational effects of drought stress.

LncRNAs were demonstrated to participate in rice short-term drought memory (Li et al. 2019). They acted as memory factors to activate phytohormone signaling genes that participate in drought memory response. The association analysis of lncRNAs and related mRNAs revealed three memory-related mRNA transcripts (TCONS_00028567, OS02T0626200-01 and OS04T0412225-00) that participate in different pathways. In Switchgrass, the levels of lncRNAs targeting the biosynthesis of ABA and trehalose increased in both first and second drought cycles, but lncRNAs regulating ethylene signaling were suppressed in the second cycle, thereby preventing leaf senescence and supporting plant development (Zhang et al. 2018).

### Transcription factors and transcriptional regulation during recurrent drought episodes

Accumulation of transcription factors (TFs) has been shown to be another possible drought memory mechanism in plants (Ding et al. 2012). For example, the transcript and protein levels found for ABF TFs indicated that ABF3 and ABF4 exhibited transcriptional memory behavior although a marginally increased protein levels in response to repeated drought stress (Virlouvet et al. 2014). In a study of epigenetic signatures of stress adaptation using *Zea mays*, Forestan et al. (2020) reveal upregulation of well-characterized transcription factors (TFs) including AP2/EREBP, NAC and WRKY families 7 days following drought recovery.

## Gene expression regulation link to physiological, biochemical and morphological responses during repeated drought stress

**Fig. 3:**
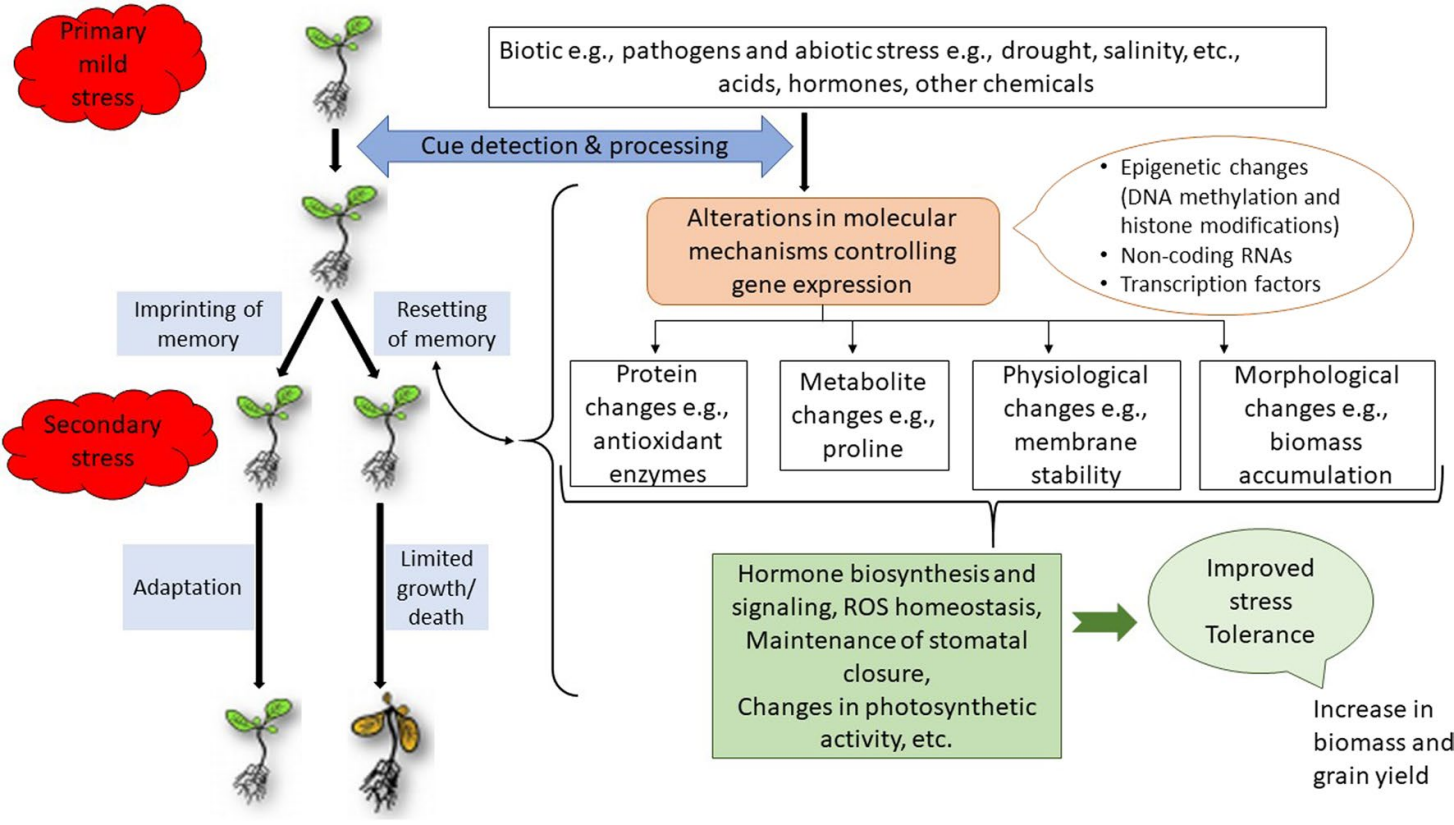
An overview of stress memory. Molecular and physiological network of drought stress response (Wojtyla et al. 2020).* ROS*, Reactive oxygen species

Gene regulatory networks involved in plants response to drought stress have been studied by examining the genes associated with drought responses, which encode regulatory and functional proteins like transcription factors (Shinozaki and Yamaguchi-Shinozaki 2007; Fujita et al. 2011; Osakabe et al. 2014). Transcriptional reprogramming is a regular aspect of the primed state (Godwin and Farrona 2020). Beyond gene expression control, other aspects have been considered in the study of plant response to reiterated stress including changes in other OMICS approaches like proteomics and metabolomics. A system–biology approach revealed that transcriptional memory correlate with physiological parameters, thereby translating into physiological memory (Virlouvet et al. 2018). In this study, 164 genes classified into four categories related to ABA biosynthesis, stomatal regulation, photosynthesis and pigments pathways were found to encode known drought stress-associated proteins. Taken together, transcripts, proteins and metabolites form interconnected, dynamic networks that mediate drought stress memory in plants (Fig. 3).

On the one hand, some drought stress-responsive genes have been shown to display regulation at transcript level that are significantly different under repeated drought exposures to the responses during their first drought contact (Ding et al. 2012; D’Urso & Brickner 2014). Memory genes according to Ding et al. (2013), are those genes that show altered responses in a subsequent stress different from the non-memory genes that remain unaltered after each round of stress. Comparable to this definition, Forestan et al. (2020) refer transcriptional memory genes, as genes with stable transcriptional changes that persist after drought recovery. Therefore, transcriptional stress memory is said to be evident when there are sustained alterations in activation or repression of genes or from a changed response following a second cue (Lämke and Bäurle 2017). On the other hand, to optimize growth and reproduction in recurrently varying environments, plants have been shown to exhibit a drought stress memory on the physiological level to reduce water loss, reduce cellular oxidative stress by maintaining reactive oxygen species (ROS) homeostasis, reduce membrane damage, reduce inhibition of enzyme activity, increase CO2 assimilation, alter photosynthetic rates and change the general morphology (Fleta-Soriano and Munné-Bosch 2016; Abid et al. 2018). Our focus here is to review transcriptional memory responses in which production of increased levels of transcript and/or enhanced repression has been shown in memory genes upon recurrent drought exposure and their association with physiological, biochemical and morphological responses during repeated drought.

### Alterations in photosynthesis and photorespiration

Alterations in photosynthesis and photorespiration mechanisms have been emphasized in different studies tackling drought memory. Generally, damage to the basic organization structure of the plant negatively affects many metabolic processes, carbon assimilation and the photosynthetic apparatus. However, priming has been shown to induce a better maintenance of photosynthetic efficiency during recurrent drought stress. Drought priming of *Triticum aestivum* L. (Abid et al. 2016; 2017) and coffee (Menezes-Silva et al. 2017) led to photosynthetic efficiency and increased Ribulose 1,5-bisphosphate carboxylase/oxygenase (Rubisco) during later stress. Wang et al. (2014) also indicated that drought primed plants before anthesis accumulated more proteins such as Rubisco small subunit, Rubisco activase and ascorbate peroxidase when subjected to another drought stress after anthesis. The propagules from drought-stressed sugarcane plants displayed increased photosynthetic water-use efficiency as well as quicker photosynthesis recovery following rehydration (Marcos et al. 2018a; b). Concurrently, drought memory genes have been by Virlouvet et al. (2018) related to photosynthesis. For example, the identified Calvin-Benson-Basham Cycle, NANDP-Me-type, NAD-ME type, PEPC, PEPCK enzyme type and PEPC kinase memory genes encode proteins that play a role in light harvesting, non-photochemical quenching, energy transfer and general photosynthesis. Memory gene that encode a chloroplast ATP synthase was down-regulated in the second stress to achieve protection of the photosynthetic apparatus.

Oñate et al. (2011) observed that when they subjected *Urtica dioica* L. in a combination of water and nutrient scarcity during juvenile stage, mature leaves revealed improved drought tolerance through modulation of chlorophyll levels during a second stress at reproductive stage. Altered chlorophyll content during subsequent encounter was also observed by Abid et al. (2016; 2017) in *Triticum aestivum* L*.* Indeed, among the 13 pigment memory genes noted by Virlouvet et al. (2018), two chlorophyll biosynthesis genes were down-regulated while two chlorophyll degradation genes were up-regulated in the second stress encounter. Although situation differ between plants based on the sink–source relationships during stress, a first water stress can improve plant response to a succeeding stress by diminishing the impact of the second stress on plant photosynthesis and energy mechanisms, thus supporting a better carbon status (Jacques et al. 2021).

### Alterations in cell integrity, osmotic and plant water status

Hormones, especially phytohormones, play important roles in the regulation of different processes of plant adaptation to drought environments by modifying cellular functions at molecular levels through diverse cell signaling (Yadav et al. 2021; Iqbal et al. 2022). Abscisic acid (ABA) is a phytohormone that during drought conditions, regulates Ca^2+^ in the guard cells to induce stomatal closure, thereby preventing water loss (Ali et al. 2020). During repeated drought exposures on *Arabidopsis thaliana* L.*,* transcriptional stress memory was displayed by an increased transcription rate and increased levels of transcripts of ABA-inducible *RAB18* (Ding et al. 2012, 2014). The transcripts accumulated progressively in every subsequent drought treatment. In their study, Forestan et al. (2020) noted higher expression levels of genes that speed up ABA biosyntheis steps (*ZEP1*, four *NCEDs* and two *AOs*) indicating stable transcriptional changes that persist after drought recovery and thereby transcriptional memory. The expression levels of ABA and jasmonic acid (JA)-related genes changed significantly in rice during the first drought exposure and the levels were stably maintained following several rounds of treatment (Li et al. 2019).

Higher ABA levels in primed wheat plants under drought stress were associated with improved tolerance to drought that occurred later during grain filling stage and subsequently to higher grain yield compared to the non-primed wheat plants (Wang et al. 2015). Fleta-Soriano et al. (2015) indicated drought memory mediated by modification in ABA by showing that the levels were raised under drought conditions if there was a previous drought exposure on the plant. Moreover, analysis of plant hormone levels in *Aptena cordifolia* L. exposed to reiterated drought revealed that Gibberelin acid went down during the first exposure and remained so in the second one, while ABA was observed to be higher in double-stressed plants compared to single-stressed plants (Fleta-Soriano et al. 2015).

Using an RNA-seq approach to investigate how *Coffea canephora* L. responded to subsequent drought, Guedes et al. (2018) were able to identify differentially expressed genes (DEG) in tolerant and sensitive clones. The findings illustrated that in the tolerant plants acclimatized to multiple drought episodes, memory genes involved in ABA pathways were identified. On the other hand, the sensitive clones were associated with memory genes that triggered an oxidative stress response that probably led to programmed death upon exposure to multiple episodes of drought. The observed transcriptional memory in tolerant and sensitive plant genes suggests the ability of the plants to opt to a mechanism to remember genes that should undergo modulation upon drought stress exposure.

An increase in expression of key ABA biosynthesis modulators including 9-CIS-EPOXYCAROTENOID DIOXYGENASE 3 (*NCED3*) and ALDEHYDE OXIDASE 3 (*AAO3*) has been indicated in previously stressed plants during recovery phase to reduce transpiration in an event of a subsequent stress attack (Virlouvet and Fromm 2015). This locus encodes a vital enzyme in the ABA biosynthesis pathway and performs an important role in signaling in drought stress. As a result of increase in the transcription of many ABA-induced genes in response to repeated drought episodes, plants reduce rates of transpiration by mediating guard cell-specific stomatal memory to keep up the leaf water content (Ding et al. 2012, 2013; Virlouvet and Fromm 2015).

A large proportion of drought memory genes in maize was by Ding et al. (2014) shown to encode for proteins associated in membrane integrity functions including dehydrins, regulators of water and potassium uptake and transport and transmembrane transporters for inorganic phosphate and sucrose. In this regard, plants that had been exposed to repeated periods of both droughts and recovery periods displayed higher retention of leaf water, reduced wilting and increased tolerance to terminal drought stress when compared to plants experiencing the stress for the first time (Jakab et al. 2005; Maseda & Fernández 2006; Ding et al. 2012; Ramírez et al. 2015). The increase in root water was also discovered in multi-generationally stressed sugarcane plants (Marcos et al. 2018b). Seedlings from drought-stressed seeds also displayed reduced membrane damage and increased water retention than the controls (Selote & Khanna-Chopra 2006, 2010; Wang et al. 2018).

Osmotic adjustment for water status maintenance is implicated in water stress plant memory (Jacques et al. 2021). Proline, an amino acid, has been shown to be a critical component of plant drought tolerance due to its role as an osmolyte. Menezes-Silva et al. (2017) reported that plants exposed to multiple drought events adapted to future stress due to the expression of trainable genes related to drought tolerance, which were associated with a deep metabolite reprogramming with concordant adjustments in central metabolic processes. Transcription memory of Δ1-pyrroline-5-carboxylate synthetase 1 (*P5CS1*) and the gene encoding of the proline biosynthetic enzyme were found to be critical in drought stress memory in rice. There was an induction of expression of *LOC_Os01g62900* and *LOC_Os05g38150*, which are *P5CS1* homologous after the initial drought stress and reached a peak during the rewatering, and then stayed constant throughout the succeeding drought stress treatment, corresponding to the level of free proline concentration (Li et al. 2019). Alves et al. (2020) also noted that proline levels in *Dipteryx alata* L. plants rose significantly following recurring drought stress. Its accumulation in the second drought exposure was also reported in peanut plants by Qin et al. (2021). However, Leufen et al. (2016) and Nguyen et al. (2020) revealed lower proline concentrations in sugar beet plants and soybeans, respectively, in the second and third drought stress episodes compared to the first stress.

Memory DMRs also regulated alpha-linolenic acid metabolism, linoleic acid metabolism, biosynthesis of amino acids, glycerophospholipid metabolism, cysteine and methionine metabolism and lysine biosynthesis pathways. Alves et al. (2020) had observed alterations in primary metabolism in *Dipteryx alata* L. plants, especially in osmoprotectants including in sugar, organic acids and amino acid levels. There were significant increases in sucrose, fructose and glucose levels in primed plants. Organic acids like citrate, fumarate, threonic acid and palmitic acid increased their levels in response to successive drought cycles. Amino acids, including glycine, histidine, alanine, GABA and tryptophan increased in plants exposed to three cycles of drought compared to those that experienced just one stress event. Oñate et al. (2011) also observed modulation of malondialdehyde (MDA) in prestressed *Urtica dioica* L. Plants. These findings indicate that past stress exposure determined the response of mature plant as these plants showed acclimation to subsequent stress.

### Key proteomic cues and drought stress memory

The general abundance and activity of proteins regulate changes in metabolic pathway activities, thereby influencing metabolite levels. Posttranscriptional regulation through changed protein abundances is an important mechanism of response to stress events, and proteomic analysis under repeated drought revealed an increased abundance of proteins (Alves et al. 2020; Auler et al. 2021a, b; Ding et al. 2013; Schulze et al. 2021). Recently, Schulze et al. (2021) have examined the proteome profiling of recurrent drought events in maize and related it to stress memory responses. The authors found overrepresentation of heat-shock proteins, ribosomal proteins, starch metabolism proteins and proteins involved in photosynthesis photophosphorylation during the first stress encounter. While rewatering recovered these proteins to basal levels, ribosomal proteins remained elevated. The second cycle of drought exposure resulted in abundances in ribosomal, galactolipid synthesis, gluconeogenesis, photophosphorylation and lipid degradation proteins but not heat shock proteins. However, Ding et al. (2013) indicated downregulation of memory genes encoding ribosomal, chloroplast and photosynthetic proteins that are involved in ribosome structure, amino acid biosynthesis and photosynthesis, in addition to memory genes that encode for thylakoid membrane-associated proteins in *Arabidopsis thaliana*. Repeated drought cycles in *D. alata* seedlings led to substantial increase in the activity of superoxide dismutases (*SOD*), pyruvate oxidase (Pox) and glutathione reductase (*GR*), which were not activated by a single drought event (Alves et al. 2020). In rice, Auler et al. (2021a) report decrease in the expression of genes that encode D1and D2 proteins of reaction center of the PSII due to a single drought stress exposure, but double drought stress increased their expression. Rehydration caused the genes to portray an expression level equivalent to that of the control plants. *TRITD1Av1G156270* gene coding for late embryogenesis abundant (LEA) proteins showed variable memory responses in rice and wheat (Sadder et al. 2022). Kim et al. (2020) observed that genes encoding protein phosphatase 2C (PP2C) family proteins and LEA proteins were differentially induced. A number of ABA- and ethylene-responsive genes encoding a putative ABA 8ʹ-hydroxylase, ABA-responsive protein-related and osmotin 34 were highly upregulated under the second drought conditions in soybeans. Comparative proteomics in guard cells between rice plants exposed only once and those with recurrent drought stress exposures at vegetative or/and reproductive stages identified 12 drought-responsive proteins that belonged to the photosynthetic pathway, oxidative stress response and stress signaling such as glucagon-like peptide-1 (GLP-1), glutathione-S-transferase (GST), SOD and those related to protein processing such as small heat-shock proteins in roots (Auler et al. 2021b). Interestingly, the abundance of proteins such as endo-1,3-beta-glucosidase, peroxidase, S-adenosylmethionine (SAMS) and malate dehydrogenase (MDH) significantly increased in roots or leaves depending on the rice genotype. Qin et al. (2021) observed a rapid increase in the expression of Arachis hypogaea abscisic acid transporter like-1 (AhATL1) protein and its levels in the second recovery periods following drought exposure. In turn, the overexpression of AhATL1 raised ABA concentrations and altered the post-response gene type into memory gene type, thereby enhancing the drought tolerance and ability to recover. Generally, these authors concluded that there were changes in protein abundance according to single or repeated drought episodes affecting many pathways in plant.

### ROS metabolism cues and drought stress memory

One of the usual consequences of drought stress is the production of ROS in the different cellular compartments, including the peroxisomes, the chloroplasts and the mitochondria. ROS includes singlet oxygen (^1^O_2_), superoxide radical (O_2_^•−^), hydroxyl radical (^•^OH) and hydrogen peroxide (H_2_O_2_) (Hasanuzzaman et al. 2020). Its overproduction results in the peroxidation of cellular membrane lipids and degradation of enzyme proteins and nucleic acids (Li and Liu 2016). To alleviate the effect of ROS, plants induce higher antioxidant enzyme activities and higher expression of their related genes, thereby conferring drought stress tolerance and adaptation (Hou et al. 2021). According to Lukić et al. (2023), the anti-oxidative system plays a crucial role in forming a plant stress drought memory through changes in the activity pattern of anti-oxidative enzymes like SOD and peroxidase (POD) as well as non-enzymatic anti-oxidative defense. The authors reported that in *Alopecurus pratensis* L. both enzymes were upregulated in drought treated offspring if the parents were also stressed. Similarly, Lukić et al. (2020, 2023) and Liu et al. (2022b) have pointed out that upregulation of the anti-oxidative system is one of the major mechanisms that mediate transgenerational drought stress memory. In their study, Lukić et al. (2023) found reduced H_2_O_2_ concentrations in drought-exposed offspring of drought-exposed parents due to increased activity of Catalase (CAT) and POX that converts H_2_O_2_ to oxygen and water. The upregulation of Superoxide SOD activity and removal of superoxide anion radicals in drought-exposed offspring of drought-exposed parents subsequently resulted to a decrease in oxidative stress levels. Moreover, malondialdehyde levels under transgenerational drought priming could be caused by increased chelation of hazardous ferrous ions that initiate lipid synthesis and the formation of MDA. Menconi et al. (1995) uncovered that two drought periods on wheat obtained by withholding water and rewatering at the end of the first period during seedling stage resulted in improved scavenging of H_2_O_2_ and control of ROS levels. A second drought stress encounter following recovery period in wheat plant revealed the enhancement of dehydroascorbate reductase, glutathione reductase and ascorbate peroxidase (Menconi et al. 1995). Correspondingly, Li et al. (2015) reported low concentrations of H_2_O_2_ in wheat leaves if drought priming was done, which could be explained by the high levels of glutathione peroxidase (GPx) in the same plants. A transformed cell structure and the expression of genes mainly encoding proteins related to redox enzymes like APX have been observed in primed plants compared with non-primed plants under drought during grain filling (Wang et al. 2014). The authors postulate that the higher APX activity in primed plants contribute to improve ROS scavenging capacity, to reduce lipid peroxidation in response to a later stress. Wang et al. (2018) found out that the O_2_^•−^ release rate and H_2_O_2_ concentration of wheat flag leaves were significantly increased under drought stress, while they were less affected by drought in the primed plants than in the non-primed plants. Moreover, the authors reported that the activities of antioxidant enzymes like SOD, CAT and APX were increased significantly by drought stress and were much higher in the primed plants than in the non-primed plants. GPX activity was much higher in the primed plants under a second drought encounter. However, only APX gene expression was consistent with its activity levels. Primed rice seedlings displayed increased POX and SOD activity to dissuade the harmful effects caused by oxidative damage in response to subsequent drought stress (Li et al. 2011). According to Yang et al., (2021), when compared with unprimed control, the primed plants showed lower CAT activity, whereas increasing the activity of SOD fivefold. In *Nicotiana tabacum* L.*,* POD activity was linked to reduced H_2_O_2_ levels in primed plants under drought treatment (Khan et al. 2020). Moreover, transcriptional levels of related genes CAT, APX1 and GR2 were revealed in drought-hardened treatment against drought stress. The expression levels of these genes were considerably increased in drought primed plants in comparison with control, and the expression of these genes was more pronounced in T3 plants than other treatments. In *Glycine max* L.*, Zea mays* L. and *Arabidopsis thaliana* L., drought memory genes that encode proteins involved in protective roles including dehydrins and chaperones were discovered (Ding et al. 2012, 2014). Synchronously, KEGG enrichment analysis results showed that the memory DMRs were involved in sesquiterpenoids, triterpenoid and phenylpropanoid biosynthesis and arginine metabolism pathways (Kou et al. 2021). These results suggest that previous drought events modified ROS scavenging systems. Defensive and detoxifying functions are important for plant stress memory since they diminish the impact of drought-induced oxidative stress by sustaining cellular metabolism (Jacques et al. 2021).

### Morphological adjustments

Plant morphological characteristics are the most valuable tools in monitoring responses to stressors as they can reveal underlying factors that produce changes in plant conditions. Nosalewicz et al. (2016) have reported the transgenerational effect of severe drought stress on shoots and roots of barley (*Hordeum vulgare* L.). The study revealed that the progeny, whose parental generation was also subjected to drought, showed adaptive morphological alterations such as increased root-to-shoot ratio when compared to the progeny of parental plants that had not been subjected to drought conditions. Backhaus et al. (2014) also reported production of higher amounts of above the ground biomass if there was a pre-exposure to drought when compared to the controls without a previous drought encounter. In agreement with these findings, Marcos et al. (2018b) observed that the plants stored information from the previous stressful events, which led the sugarcane plants that were drought-stressed three times to have increased root dry matter.

## Conclusions and future directions for research on drought stress memory and its application in breeding

Changes in the epigenome, transcriptome, proteome and metabolome upon stress encounter confers stress memory, which enable enhanced responses to future stress exposure in plants. Uncovering the potential of this phenomena in crops and how best this discovery can be used in plant breeding programs require an integrated approach. Taken together, the reviewed studies here provide results that point to high variation of species and/ or genotypes specificity to drought stress memory responses. Such studies provide new opportunities for plant breeders and researchers in exploiting different memory capabilities in plants to develop new cultivars in the face of changing climates.

The discovery that plants can memorize past stressful events and pass it to their progeny offers an opportunity to adjust plants’ epigenetic architecture and find out how and which genes are expressed to adjust the growth of plant to adapt to the environment. Indeed, exposure to a priming agent could activate a gene or a set of genes. However, instead of reverting to the transcriptionally silent state once the stimulus is removed, an epigenetic modification could perhaps be left, keeping the region in a ‘permissive’ state. As a result, there is a possibility for quicker and more potent responses to subsequent attacks. This discovery can offer a non-traditional approach to breeding because gene networks that are targeted by this manipulation can be identified without altering the genotype. If a memory gene is identified, it can be regulated to make the plant behave as if it is experiencing the stress, and the mechanisms related to stress tolerance are elicited all the time through the expression of other related genes.

The nature of experiments carried out in the study of stress memory should be assessed for success and applicability. Usually, the recovery period following an initial stress is when stress information is integrated and therefore is crucial for the reinforcement of correct stress memory. In addition, experiments should incorporate different priming stages in the life span of a given crop to evaluate which stage induces most pronounced beneficial impacts. Moreover, it would be necessary to prepare, grow and multiply the seed for an experiment or a selection procedure in exactly the same way, so that the memory does not affect the outcome of the experiment or selection. This would also guarantee that memory effects between experiments are duplicated. Validation is also essential when transforming laboratory or controlled experiment information to the field. As depicted in this review, various imprints including hormones (ABA, Gibberelin acid and JA), enzymes (antioxidants such ascorbate peroxidase) and metabolites like proline are strong causes or consequences of plant memory response. Based on these studies, we propose that investigations on their concentrations during recurrent drought episodes, associated memory genes, as well as related epigenetic marks be carried out. Lastly, there are variable results regarding the usefulness of priming and persistence of the discovered drought stress memory. Therefore, further research is needed to explore the influence of priming on plant population and community structures as it involves plant performances and reproductive success. Researchers should in future also find out how the positive stress memory effects can be increased and prolonged.
